# Exploring the Therapeutic Potential of *Cordyceps* Mushroom on SARS-CoV-2 Using Virtual Screening against M^pro^ and In Vitro Validation of Cordycepin

**DOI:** 10.4014/jmb.2411.11063

**Published:** 2025-03-26

**Authors:** Mohammad Hassan Baig, Ayman Turk, Preeti Vishwakarma, Yun Seong Jo, Jae-June Dong, Dae Hee Lee, Young Guk Kim, Mi Kyeong Lee, Jae-Yong Cho

**Affiliations:** 1 Department of Family Medicine, Gangnam Severance Hospital, Yonsei University College of Medicine, Seoul 06229, Republic of Korea; 2 College of Pharmacy, Chungbuk National University, Cheongju 28160, Republic of Korea; 3 Translational Health Science & Technology Institute, National Capital Region (NCR) Biotech Science Cluster, Faridabad, India; 4 Doobon Inc., Chungbuk 28174, Republic of Korea; 5 Department of Internal Medicine, Gangnam Severance Hospital, Yonsei University College of Medicine, Seoul 06229, Republic of Korea

**Keywords:** *Cordyceps militaris*, COVID-19, cordycepin, M^pro^, MD simulation

## Abstract

Pathogenic coronavirus, including COVID-19, threatens human health, and there have been strong demands for efficient therapeutics. *Cordyceps militaris* is a medicinal mushroom that has long been used for immune enhancement, anticancer, and antiviral effects. Therefore, the inhibitory potentials of constituents of *C. militaris* against COVID-19 were analyzed using various virtual screening analyses. Among ten constituents of *C. militaris*, cordycepin, the major component, and 3’-deoxyuridine and 2’-*O*-methyl-adenosine showed strong binding affinity to M^pro^, a potential target for COVID-19 therapeutics. Considering the structure-activity relationship, nucleosides having deoxyribose and methoxyribose moiety are important for the affinity to M^pro^. Cordycepin is also bound to M^pro^ mutants, and the binding mechanisms between cordycepin and M^pro^ were investigated further by MD simulation and MM/PBSA analysis. Principal component analysis also confirmed the conformational change of M^pro^ by cordycepin, which inhibits the function of M^pro^. *In vitro*, the efficacy of cordycepin was measured using Vero cells infected with SARS-CoV-2, which showed excellent inhibition with an IC_50_ value of 29 μM. Conclusively, the constituents of *C. militaris* are expected to inhibit SARS-CoV-2 replication through binding to M^pro^. Therefore, *C. militaris* can be an essential therapeutic for coronavirus through the synergistic effect of its constituents.

## Introduction

Since the first outbreak of Coronavirus disease 2019 (COVID-19), this pandemic has caused major global health issues and threatened worldwide medical settings [1]. The risk of COVID-19 appears to be somewhat decreased compared to the beginning, but it is still threatening public health with the emergence of new variants [2].

Pathogenic coronavirus, which includes COVID-19, also contains Severe Acute Respiratory Syndrome coronavirus (SARS-CoV) and Middle East Respiratory Syndrome coronavirus (MERS-CoV) which caused SARS and MERS, respectively. COVID-19 is caused by SARS-CoV-2, the positive-sense single-stranded RNA virus [3, 4]. The viral entry of SARS-CoV-2 within the host cell is mediated by binding its surface spike glycoprotein (S protein) to the host angiotensin-converting enzyme 2 (ACE2) [5, 6]. Comprising 14 open reading frames (ORFs), the SARS-CoV-2 genome encodes polyprotein precursors (pp1a and pp1ab), four structural (spike, envelope, nucleocapsid, and membrane protein) and accessory proteins [7-9]. The two polyproteins (pp1a/pp1ab) cleave into individual nonstructural proteins to replicate the viral genome. This cleavage of pp1a/pp1ab is mediated by two main proteases, main protease (M^pro^) and papain-like protease (PL^pro^) [7]. Therefore, for the replication and infectious capacity of SARS-CoV-2, proteolytic enzymes, SARS-CoV-2 M^pro^ and SARS-CoV-2 PL^pro^ are essential and thus have become important antiviral targets [8-10]. Notably, SARS-CoV-2 M^pro^ is considered a potential target for COVID-19 therapeutics due to the difference with the human protease and the consequent side effects [10, 11].

As therapeutics for COVID-19, various agents with different mechanisms are being used [12, 13]. Among them, antiviral agent that suppress the replication of SARS-CoV-2 are among the most important therapeutic methods. Remdesivir, a representative antiviral agent, is a synthetic derivative of nucleosides and inhibits viral replication by replacing normal nucleic acids due to their structural similarity [14-16].

*Cordyceps militaris* is a parasitic fungus that develops on the bodies of insects in the wild. Cordycepin, 3'-deoxyadenosine is the major constituent and has diverse pharmacological effects, such as anticancer and immunostimulatory activity [17-19]. It has also shown antiviral activity against EBV, HIV, and influenza virus [20, 21]. Cordycepin also has drawn particular attention due to its similarity to nucleosides. It suppresses transcription by binding to adenosine receptors, eventually inhibiting proliferation [22]. Therefore, the therapeutic potential of cordycepin against COVID-19 has been investigated using computational analyses [23, 24].

*Cordyceps* mushroom is a rich source of cordycepin and also contains various components including cordycepin derivatives. Generally, the use of extract as therapeutic agents is expected to have synergistic effect of the various component [25]. It is also economically advantageous because no additional processes, such as purification, are required. In our earlier investigation, we isolated several compounds from Cordyceps, including cordycepin derivatives [26]. To the best of our knowledge, while the potential of cordycepin as a treatment for coronavirus has been studied, no studies have been conducted on other components of *C. militaris*. In this study, we attempted to analyze other constituents of *Cordyceps* against SARS-CoV-2 M^pro^ with the expectation that they would also have therapeutic potential against COVID-19. *In vitro* antiviral evaluation of the active compound was also conducted using Vero cells infected with SARS-CoV-2.

## Materials and Methods

### Reagents

Lopaminivir, chloroquine, and remdesivir were obtained from SelleckChem, Sigma-Aldrich, and MedChemExpress, respectively. An anti-SARS-CoV-2 N protein antibody was purchased from Sino Biological Inc. (China). Alexa Fluor 488 goat anti-rabbit IgG (H+L) secondary antibody and Hoechst 33342 were purchased from Molecular Probes.

### Structure Analysis of SARS-CoV-2 M^pro^

The structure of the Main Protease (M^pro^) of SARS-CoV-2 was sourced from the RCSB protein data bank (PDB ID: 6LU7) [27]. It comprises 309 amino acids. Mutant structures with single point mutations (IDs 8EHM, 8EHK, 8E26, 8E1Y, 8DZA, 8DZ6, 8DZ1, 8DGB, 8DDM, 8DDI, 8D4N, 8D4L, 7ZB8, 7ZB7, 7U29, 7U28, and 7N6N) were scrutinized.

### Virtual Screening

The structural data for the SARS-CoV-2 Main Protease (M^pro^) was obtained from the RCSB protein data bank (PDB ID: 6LU7) [28]. The structures for the selected compounds were gathered from the PubChem compound database. All the compounds were screened against M^pro^, and the compound with the highest docking score was selected.

### Molecular Dynamics Simulation

The structure of cordycepin, the highest-scoring compound in complex with M^pro^, was subjected to Molecular dynamics simulation using the Gromacs (2023.03 packages) [29]. The cubic box model was executed in the TIP3P water molecules for solvation, maintaining a 10 Å radius as its boundary, along with the CHARMM27 force field. Ligand topology was produced with SwissParam. The system was neutralized using sodium and chloride ions. The protein-ligand complex, together with water and ions, underwent energy minimization for 50,000 steps using the steepest descent method. Isothermal and isochoric equilibration was performed using Particle Mesh Ewald for long-range electrostatics for 50,000 steps. Isothermal and isobaric equilibration also employed Particle Mesh Ewald for long-range electrostatics for 50,000 steps. After two equilibrations, an MD run was carried out for 100 ns.

### Free Energy Calculation

The module MMPBSA.py was utilized to conduct MM-PBSA calculations using the AMBER software. This method computes the binding free energy (ΔG binding) grounded on the subsequent equation:

Δ*G_binding_* =Δ*G_MM (Potential energy in vaccum)_* + Δ*G_sol (solvation effects)_*,

Δ*G_MM_* =Δ*G_coulomb (electrostatic interaction)_* + Δ*G_Vdw_*,

Δ*G_sol_* =Δ*G_polar_* + Δ*G_nonpolar_*.

### Isolation of Cordycepin

Cordycepin was purified from the dried *C. militaris*, as previously reported [30]. Briefly, the dried *C. militaris* was extracted with 80% methanol for one night. The extract was evaporated under reduced pressure to give a methanolic extract. After the extract was suspended in water, column chromatography using Diaion HP-20 was conducted with the elution using a mixture of methanol and water. Cordycepin was purified from the fraction eluted with H_2_O: MeOH (80:20) by semipreparative HPLC.

### In Vitro Evaluation of Cordycepin against SARS-CoV-2

In vitro evaluation of cordycepin against SARS-CoV-2 was performed at the Institute Pasteur Korea, South Korea using the standard protocol [31, 32].

Vero cells (ATCC CCL-81) were maintained at 37°C with 5% CO_2_ in Dulbecco’s modified eagle medium (DMEM; Welgene, Korea), supplemented with 10% heat-inactivated fetal bovine serum (FBS) and 1× antibiotic-antimycotic solution (Gibco, USA). SARS-CoV-2 (βCoV/KOR/KCDC03/2020) was provided by the Korea Centers for Disease Control and Prevention (KCDC) and was propagated in Vero cells. All experiments involving live SARS-CoV-2 followed the guidelines of the Korea National Institute of Health (KNIH) using enhanced biosafety level 3 (BSL3) containment procedures at Institute Pasteur Korea approved for use by the KCDC.

To evaluate compounds, Vero cells were seeded at 1.2 × 10^4^ cells per well in DMEM, supplemented with 2% FBS and 1× antibiotic-antimycotic solution (Gibco), in 384-well plates 24 h before the experiment. Ten-point DRCs were generated, with compound concentrations ranging from 0.1 to 50 μM. Approximately 1 h after compound treatment, SARS-CoV-2 (0.0125 MOI) was infected with cells in a BSL3 facility and incubated at 37°C for 24 h. After that, the cells were fixed with 4% paraformaldehyde (PFA), and then permeabilized. The cells were then stained by treating the anti-SARS-CoV-2 nucleocapsid (N) primary antibody and the Alexa Fluor 488-conjugated goat anti-rabbit IgG secondary antibody and Hoechst 33342. Images were acquired by a Perklin Elmer Operetta (20×; USA) and analyzed by in-house developed Image Mining 3.0 (IM 3.0) plug-in software.

### DRC Analysis by Immunofluorescence

A ten-point DRC was produced for each molecule. Vero cells were plated at a density of 1.2 × 10^4^ cells per well in DMEM medium, including 2% FBS and 1% antibiotic-antimycotic solution (Gibco), on black, 384-well Clear plates (Greiner Bio-One) 24 h before the experiment. Ten-point DRCs were created, including chemical concentrations ranging from 0.1 to 50 μM. To study viral infections, the plates were moved to the BSL3 containment facility and SARS-CoV-2 was introduced with a multiplicity of infection (MOI) of 0.0125. The cells were treated with 4% PFA and examined using immunofluorescence 24 h after infection. The obtained pictures were examined using proprietary software to measure the number of cells and infection rates, and the effectiveness of the antiviral treatment was standardized against the positive (mock) and negative (0.5% DMSO) controls on each test plate. The sigmoidal dose-response models were used to fit the DRCs, using the equation: Y = bottom + (top-bottom) /[1 + (IC50/X) ^Hillslope^]. This analysis was conducted using either XLfit 4 software or Prism7.

## Results and Discussion

### Interaction between Cordyceps Compounds and M^pro^

**Fig. 1:**
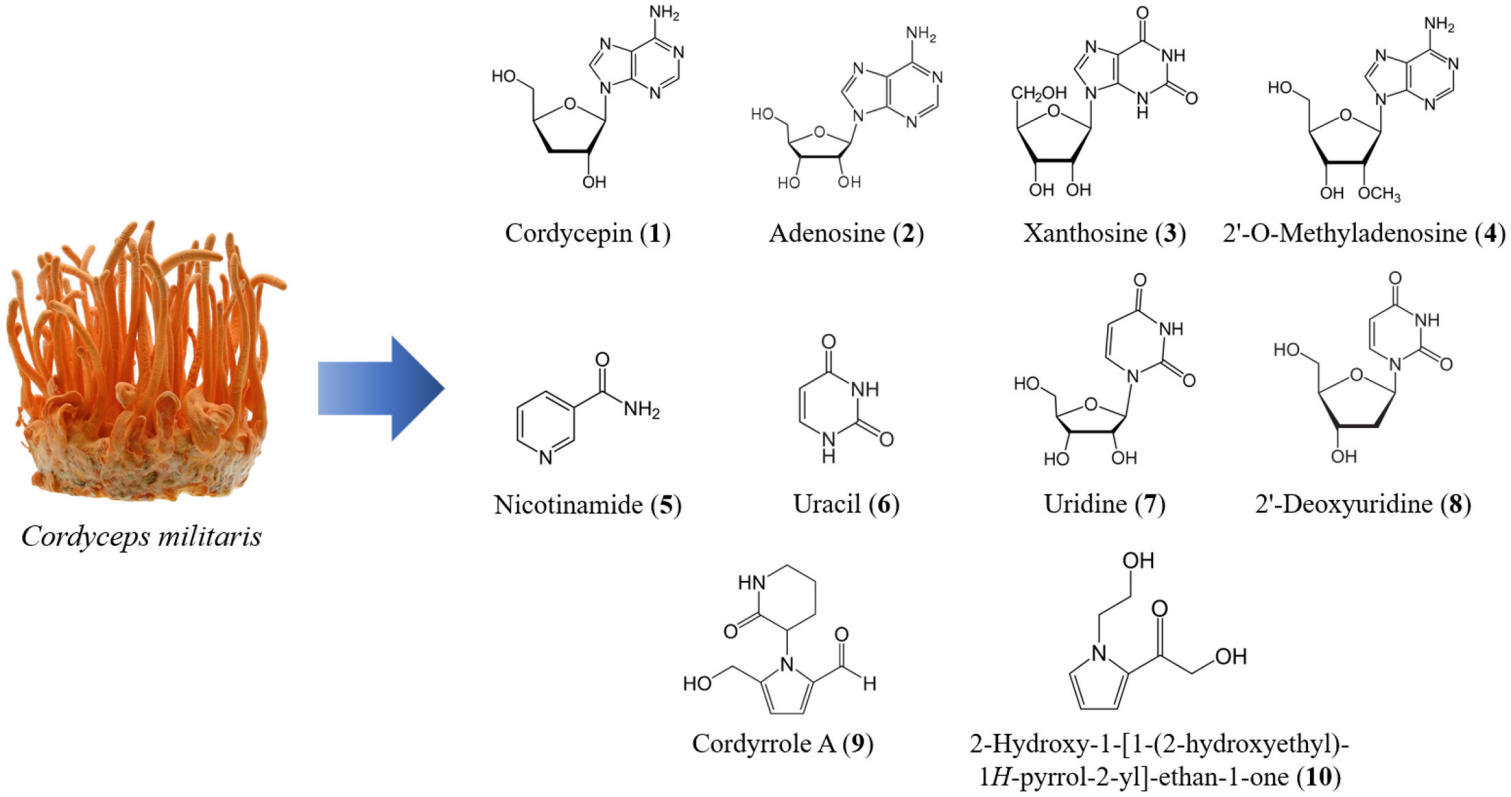
Structures of compounds from *C. militaris* that were evaluated in this study.

We initially explored the inhibitory capabilities of Cordyceps compounds against SARS-CoV-2. We analyzed the binding affinity of the compounds which we previously isolated from *C. militaris* (Fig. 1) [26] to screen out the most active compound with higher binding affinity against M^pro^.

**Fig. 2:**
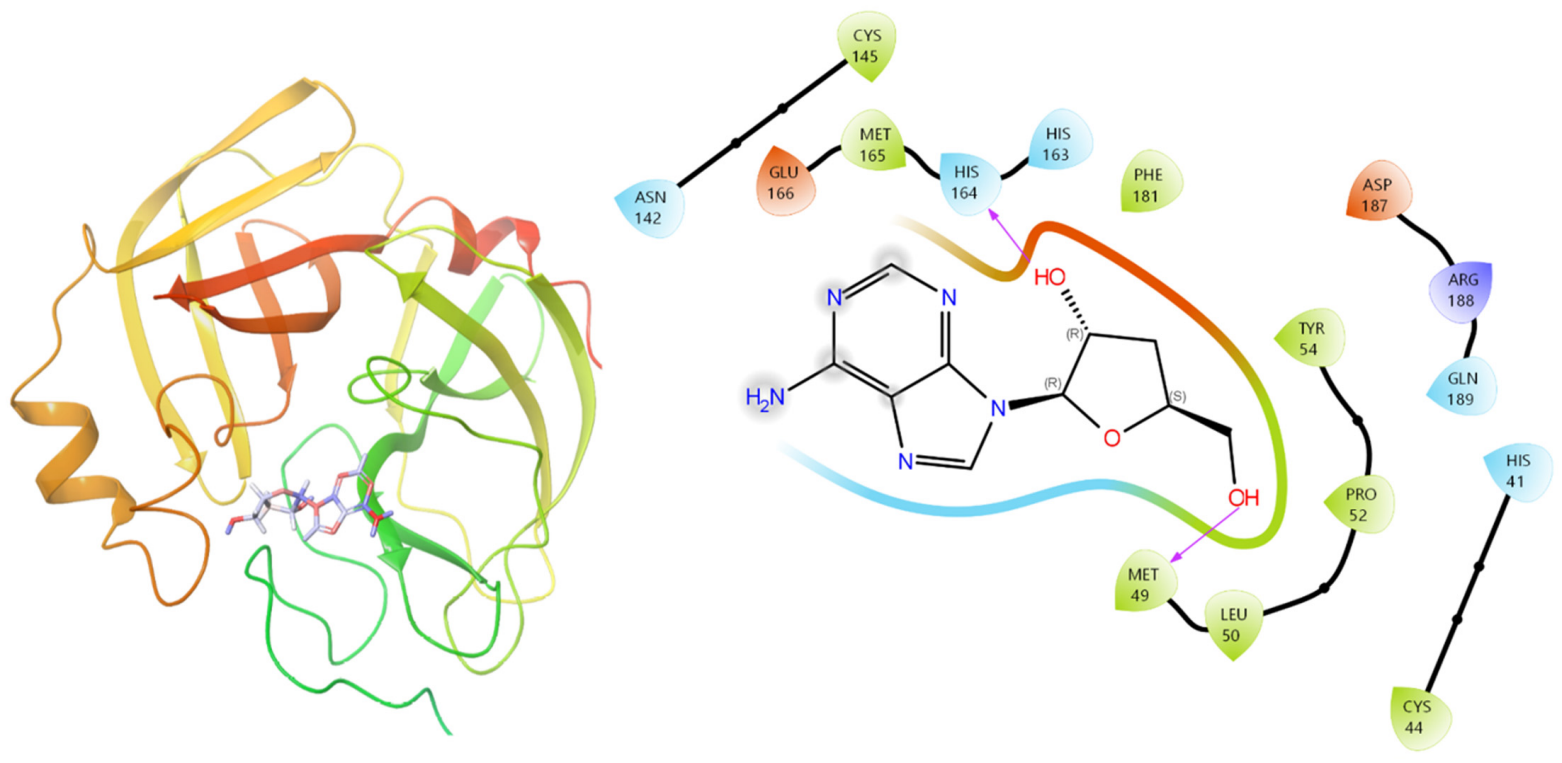
Cordycepin docked complex within the active site of M^pro^.

Among the compounds examined, cordycepin exhibited the highest docking score of -6.774, closely followed by adenosine (-6.756), and xanthosine (-6.392). 2’-*O*-Methyl-adenosine and nicotinamide also demonstrated an affinity for M^pro^, with docking scores of -6.136 and -5.981, respectively. Conversely, compounds such as uracil, uridine, 2’-deoxyuridin, cordyrrole A, and 2-hydroxy-1-[1-(2-hydroxyethyl)-1H-pyrrol-2-yl]-ethan-1-one showed comparatively weaker affinities. The study showed several active site residues, M49, P52, and R188, which facilitate the ligand binding within the active site of M^pro^, prominently involved in making hydrogen bonds with cordycepin (Fig. 2 and Table 1). The Cys-His dyad within the active site of M^pro^ has been well studied to show protease activity. Cordycepin was found to be making Pi-Alkyl interaction with H61 of this catalytic dyad. M49, a substrate union triad 33-34 member, was also found to make a hydrogen bond with cordycepin. Along with these interactions, several other residues contribute to accommodating the molecules by making hydrophobic or pi-cation interactions (Fig. 2).

Considering the chemical structures of compounds, cordycepin (**2**) and 2’-*O*-methyl-adenosine (**3**) share the same structures as adenosine (**1**) except for the removal of a hydroxy group (OH) at 3’ of ribose or the addition of methoxy group (OCH_3_) to 2’ of ribose, respectively. Xanthosine (**4**) also has a ribose moiety similar to adenosine (**1**) but differs in nucleotide moiety. All these compounds showed strong affinities to M^pro^, which suggested the importance of that ribose moiety for the affinity to M^pro^, and the increase of the affinity by removing hydroxy or adding methoxy moiety to ribose. Consistently, the compounds **7-10** without ribose moiety showed relatively weak affinities to M^pro^. These results suggested that cordycepin and other Cordyceps compounds have an inhibitory effect on M^pro^ and can be useful as therapeutics against SARS-CoV-2.

### The Binding Efficacy of Cordycepin against Mutated Forms of SARS-CoV-2 M^pro^

**Fig. 3:**
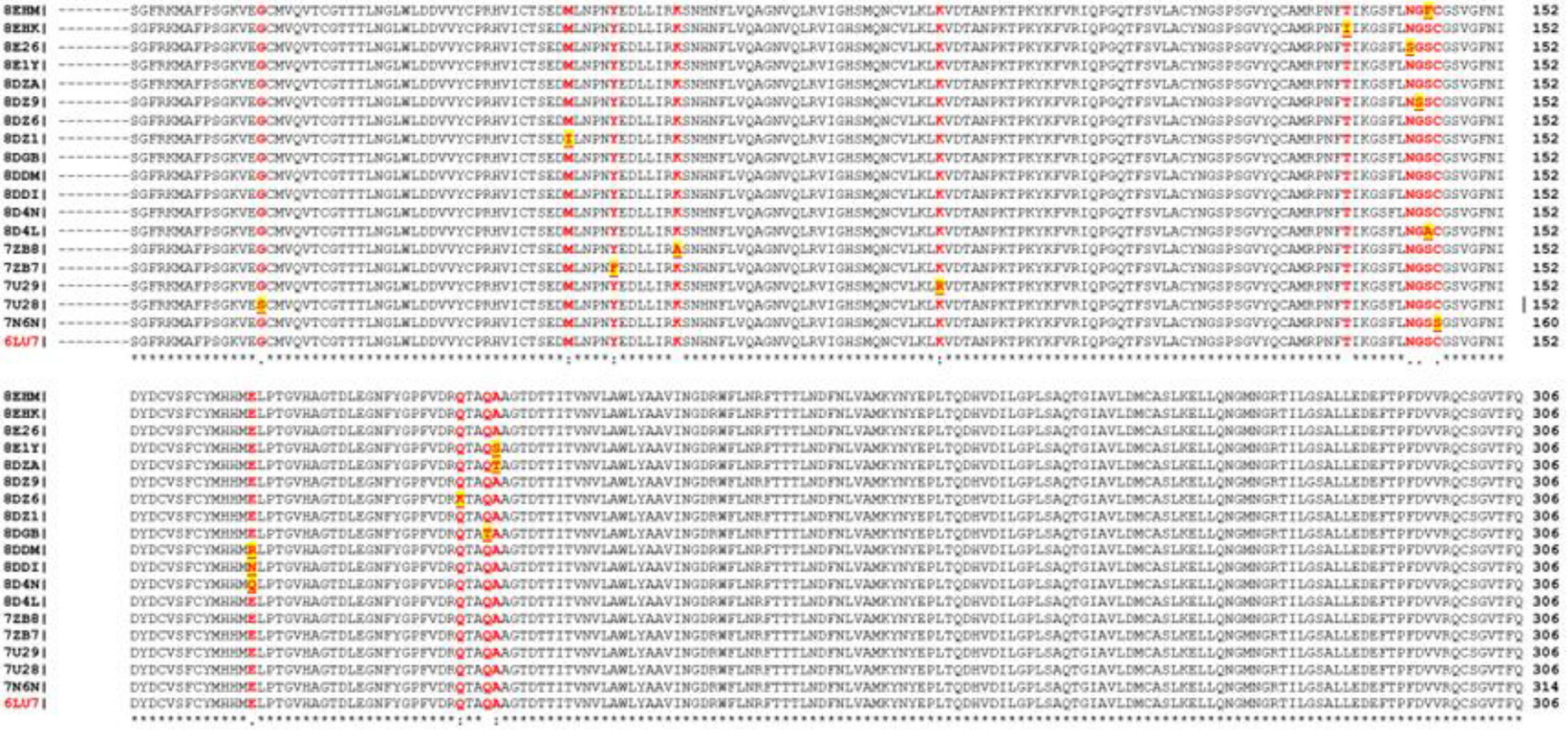
Sequence comparison among SARS-CoV-2 M^pro^ mutant protein. Eighteen distinct mutants were juxtaposed with the reference structure 6LU7. Residues highlighted in red denote mutant positions. Underlined residues represent mutant amino acids.

Given that cordycepin exhibited the highest affinity, additional research was performed to understand its action against SARS-CoV-2 M^pro^. The incidence of mutation is a standard occurrence in viral systems, which further complicates the identification of vaccine/drug candidates [33]. The emergence of mutations in SARS-CoV2 M^pro^ can potentially lead to drug resistance [34]. Thus, we further examined active site mutations within M^pro^ to verify binding affinity of cordycepin against these mutants. As depicted in Fig. 3, 10 out of the 14 high-frequency point mutations near the M^pro^ protease binding pocket were identified in the alignment of the wild and mutant sequences. Mutations M49I, Y54F, N142S, G143S, S144F/A, C145S, Q189K, Q192T, A193S/T, and E166R/N/Q were located within a 5Å distance of the ligand pocket.

**Fig. 4:**
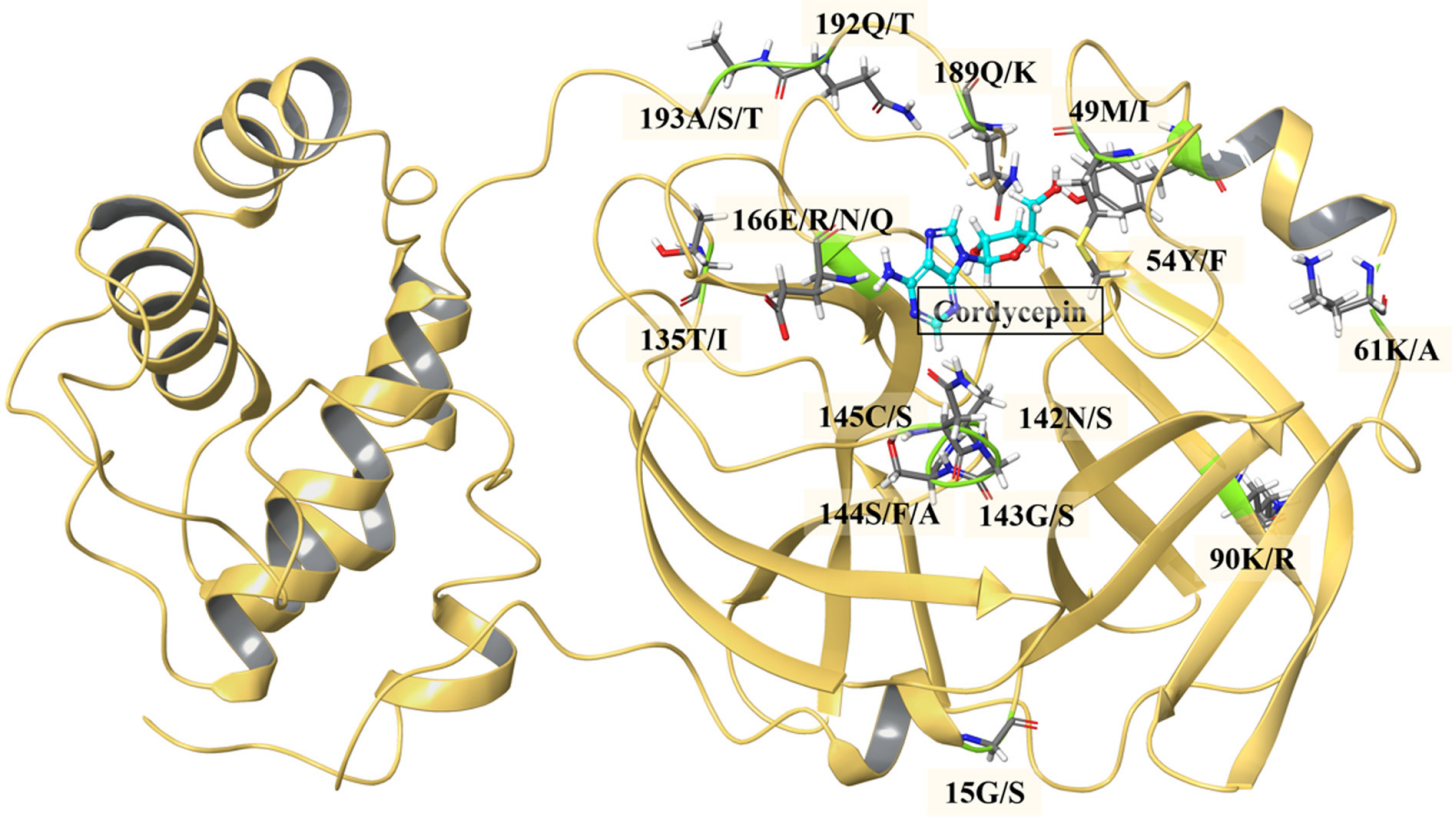
Cartoon depiction of the docked complex of cordycepin with CoV-2 M^pro^ mutant protein. The positions of the mutants are highlighted in stick forms. Cordycepin (multicolor ball and stick), is shown docked in the binding pocket.

**Fig. 5:**
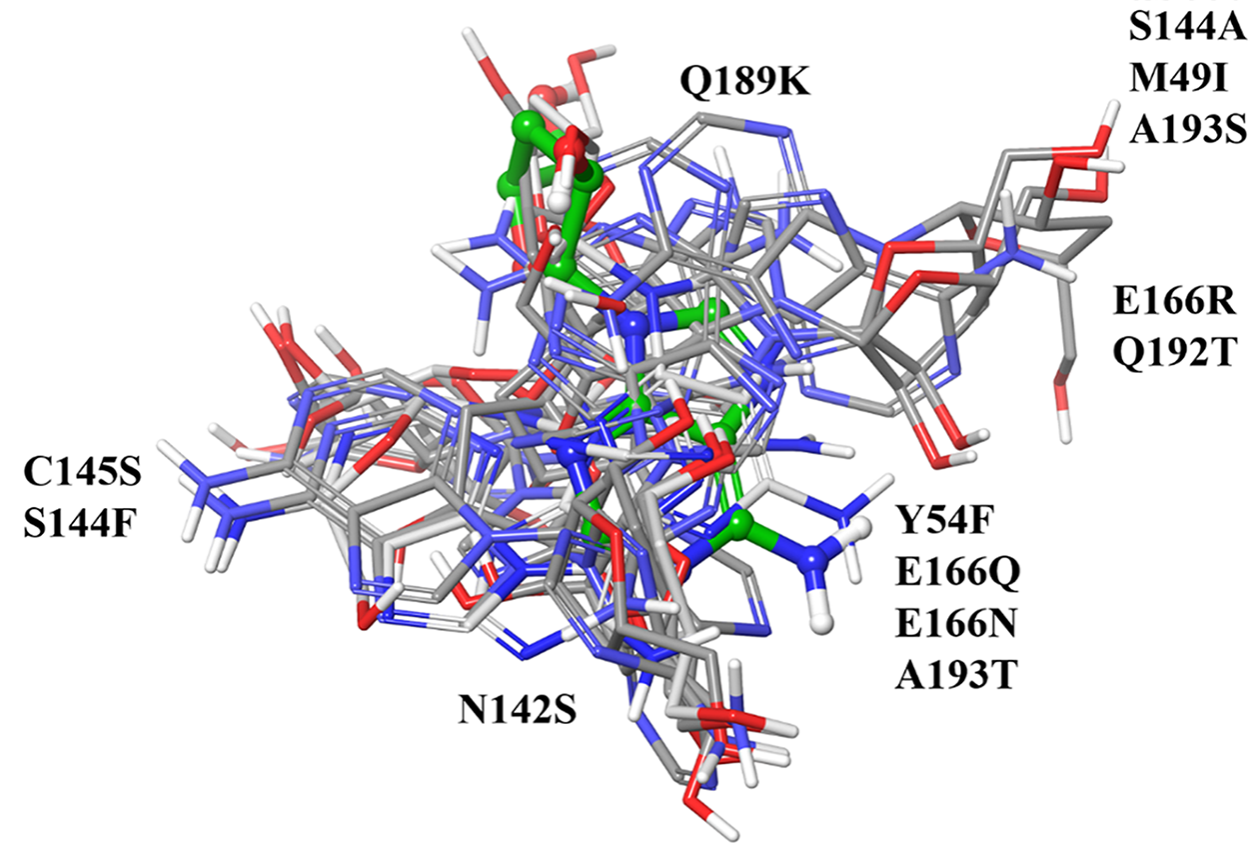
Clustering of the docked ligands in various mutants demonstrating scattered poses. The poses are indicated with representative mutant positions and residues.

We further delve into the binding potential of cordycepin against all the 14 M^pro^ mutant structures by the clustering of docking outcome poses (Fig. 4). The effect of these mutations on the ligand binding is minimal, with no significant changes in the interacting partners (Fig. 5).

### Molecular Dynamics Simulation: Probing the Interaction of Cordycepin and SARS-CoV-2 M^pro^

**Fig. 6:**
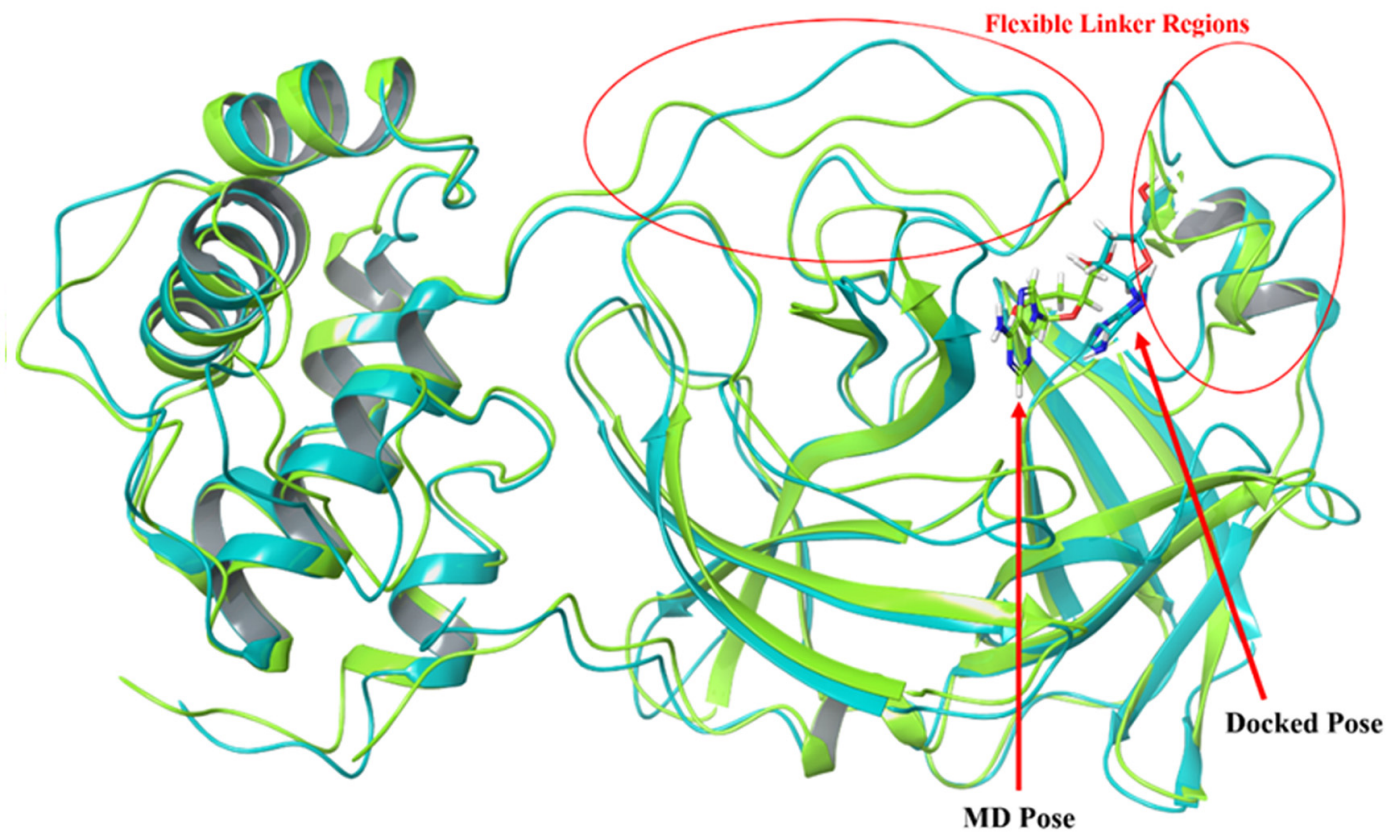
The comparative analysis of the structures of the docked and post-simulated M^Pro^-cordycepin complexes.

An MD simulation study was carried out to evaluate the degree of interaction between cordycepin and M^pro^. Both the structures of the unbound (apo) and cordycepin-bound M^pro^ were subjected to 100 ns MD simulations and assessed using various investigational parameters. The thermodynamically stable simulated complex of M^pro^ and cordycepin was then compared to the initial docked complex to comprehend the dynamics of the ligand and the secondary structure elements of the protein. As depicted in Fig. 6, a translational shift in the ligand pose within the binding pocket was noticed. Furthermore, the hyper-flexible linker region, which lies between D176-T198 and I43-S62 and surrounds the pocket, showed a varied orientation throughout the simulation period. The two hydroxy groups on the tetrahydrofuran of the ligand are more favorably oriented toward the anti-parallel β-barrel structure of the catalytic pocket.

### RMSD Evaluation of Interaction of Cordycepin with SARS-CoV-2 M^pro^

**Fig. 7:**
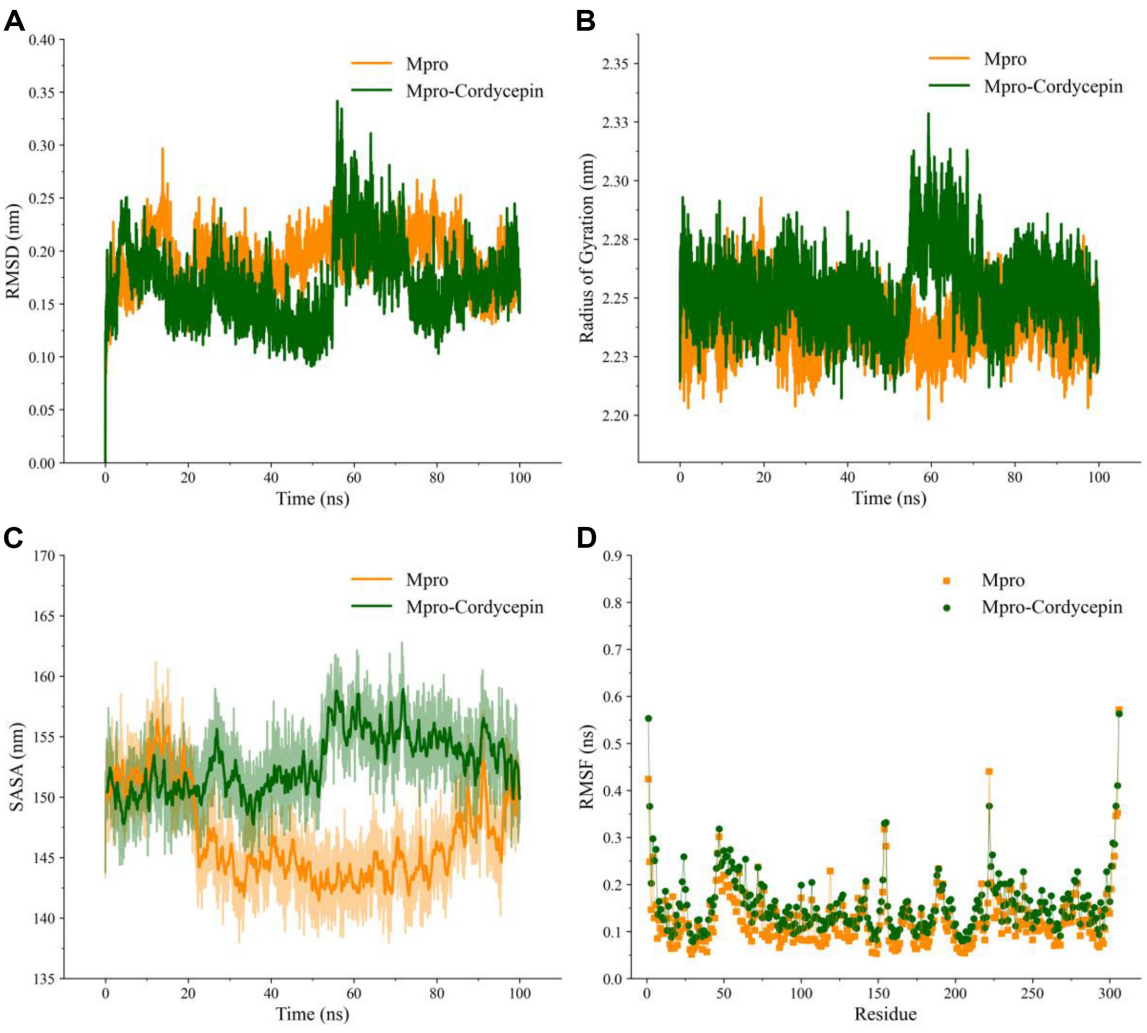
The results of molecular dynamics for the apo and cordycepin-bound M^pro^ structures over the course of the 100 ns. (**A**) The backbone RMSD of the unbound and cordycepin-bound M^pro^. (**B**) The radius of gyration, and (**C**) SASA of the unbound and cordycepin-bound M^pro^. (**D**) The root mean square fluctuation of the residues in the unbound and cordycepin-bound M^pro^ states over the 100 ns.

RMSD measures the deviation of a protein's backbone from its initial to final structural conformation. These backbone deviations during the simulation period can be used to determine the protein stability concerning its structural conformation [35, 36]. Therefore, the fluctuation in the Cα backbone of M^pro^ in the presence and absence of cordycepin was assessed by the backbone RMSD measurement. The backbone of the unbound (apo) and cordycepin-bound M^pro^ reached equilibrium conformation after 70 and 50 ns, respectively. The RMSD of the unbound and cordycepin-bound M^pro^ structures remained consistent throughout the period (Fig. 7A). Subsequently, the level of structural compactness within the cordycepin unbound and bound M^pro^ structures was determined using the radius of gyration (Rg) and SASA analysis [37]. The Rg of the Cα atoms of the unbound and cordycepin-bound M^pro^ during the 100 ns simulation time is depicted in Fig. 7B. The Rg of the cordycepin-bound M^pro^ was found to be slightly higher than that of the unbound form, indicating that the unbound M^pro^ was more stable than the cordycepin-bound form. In line with the RMSD and Rg results, the SASA plot also showed a slightly higher value for the cordycepin-bound M^pro^ structure (Fig. 7C). Additionally, the RMSF analysis revealed a distinct difference in the fluctuation of the residues between the cordycepin-bound and unbound M^pro^ conformations (Fig. 7D).

### Molecular-Mechanics Poisson-Boltzmann Surface Area (MM/PBSA) Analysis

The molecular-mechanics Poisson-Boltzmann Surface Area (MM/PBSA) method is applied to estimate the difference in the binding free energy of the complex [38]. The binding free energy is calculated by considering the vacuum potential energy, solvation-free energy (polar), and solvation-free energy (nonpolar). Polar and nonpolar solvation energy terms were estimated using the Poisson–Boltzmann equation and solvent-accessible surface area (SASA) methods [39, 40]. The Poisson–Boltzmann equation approximates the electrostatic component of biological macromolecules. It assesses the ligand-binding affinity of the protein, while the SASA method aids in identifying the surface of the protein with van der Waals contact probed by the solvent sphere [41].

The MM/PBSA result indicated the dominance of hydrophobic forces between the ligand and the target protein throughout the simulation period (Table 2). The binding free energy for cordycepin calculated by the Molecular mechanics force field was found to be relatively high at -5.39 kcal/mol, probably due to the minor presence of the polar fields in the binding pocket.

### Analyzing Cordycepin against M^pro^ through Principal Component Analysis

**Fig. 8:**
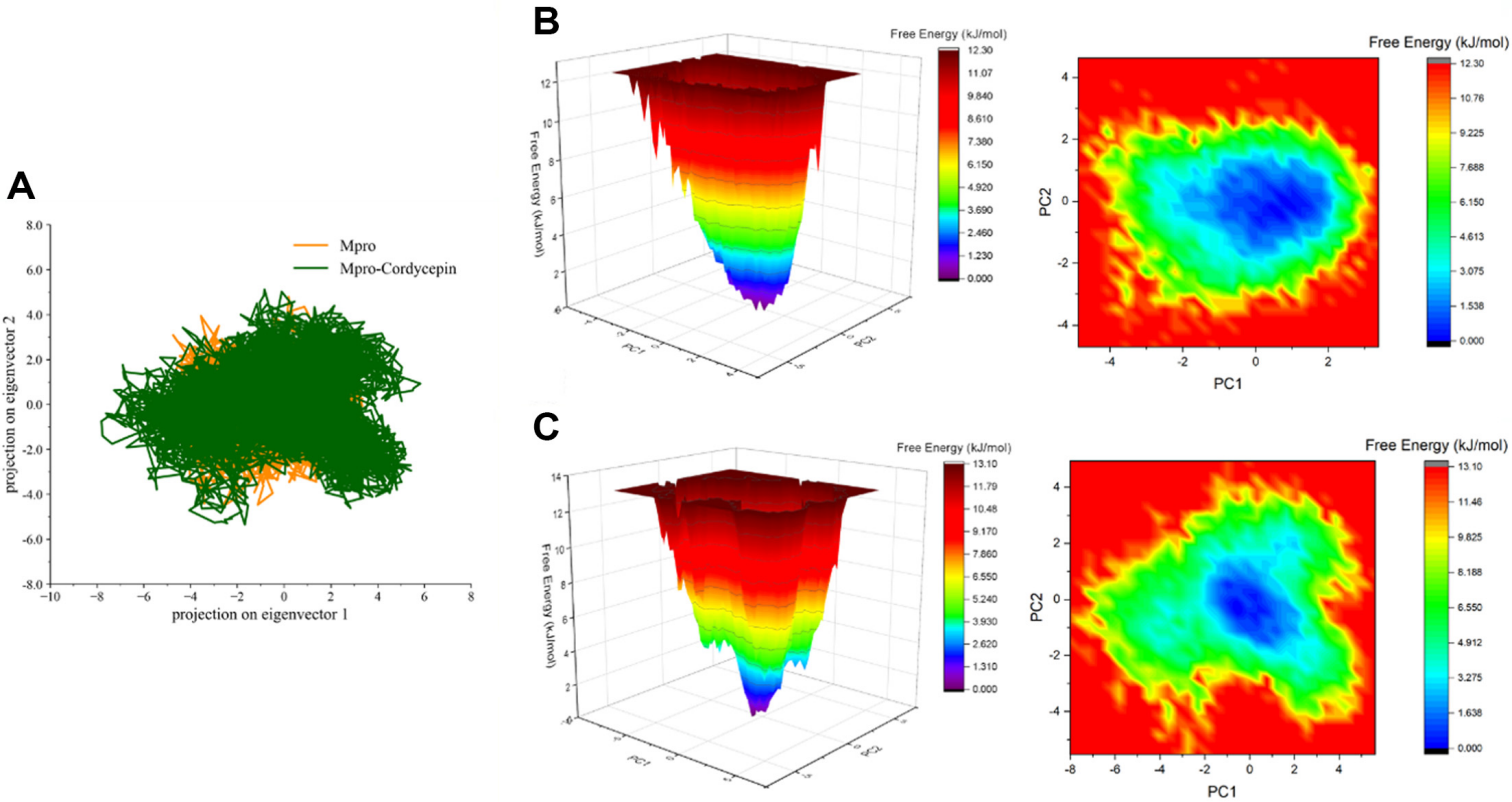
The PCA of M^pro^ in the unbound and cordycepin-bound states. Visualization of the free energy landscape for the (B) unbound and (C) cordycepin-bound M^pro^ complex.

Principal component analysis (PCA), a method that accounts for the essential dynamics, was employed to investigate the higher atomic motions patterns among all the motions within the cordycepin unbound and bound state of M^pro^. Fig. 8A shows the conformational sampling of the tertiary structure for the apo- and cordycepin-bound structures in the essential subspace along eigenvectors 1 and 2. It was noticed from PC1 and PC2 projections that the cordycepin bound structure shows a less compact cluster of stable states. The analysis shows that the cordycepin bound M^pro^ covers a wide range of phase spaces (higher-level internal motions). This study indicates that the apo form of M^pro^ has comparatively fewer internal motions, indicating higher stiffness and stability of this structure.

Additionally, we plotted the free energy landscapes to understand better the apo- and cordycepin-bound structures of M^pro^ (Fig. 8B and 8C). The knowledge of the free energy landscape of a protein offers the possibility of characterizing essential structural aspects, including its stability, folding pattern, and molecular recognition [42]. Here, we analyzed for any differences in the protein-folding patterns in both structures (apo and cordycepin bound). A slight deviation in the projection of free energy was noticed with relatively stable conformation and energetic favorability for the apo form of M^pro^ compared to the cordycepin-bound complex. All the investigations indicate that the binding of cordycepin with M^pro^ slightly perturbs its conformation, subsequently inhibiting its activity.

### In Vitro Evaluation of Cordycepin Using SARS-CoV-2 Infected Vero Cells

**Fig. 9:**
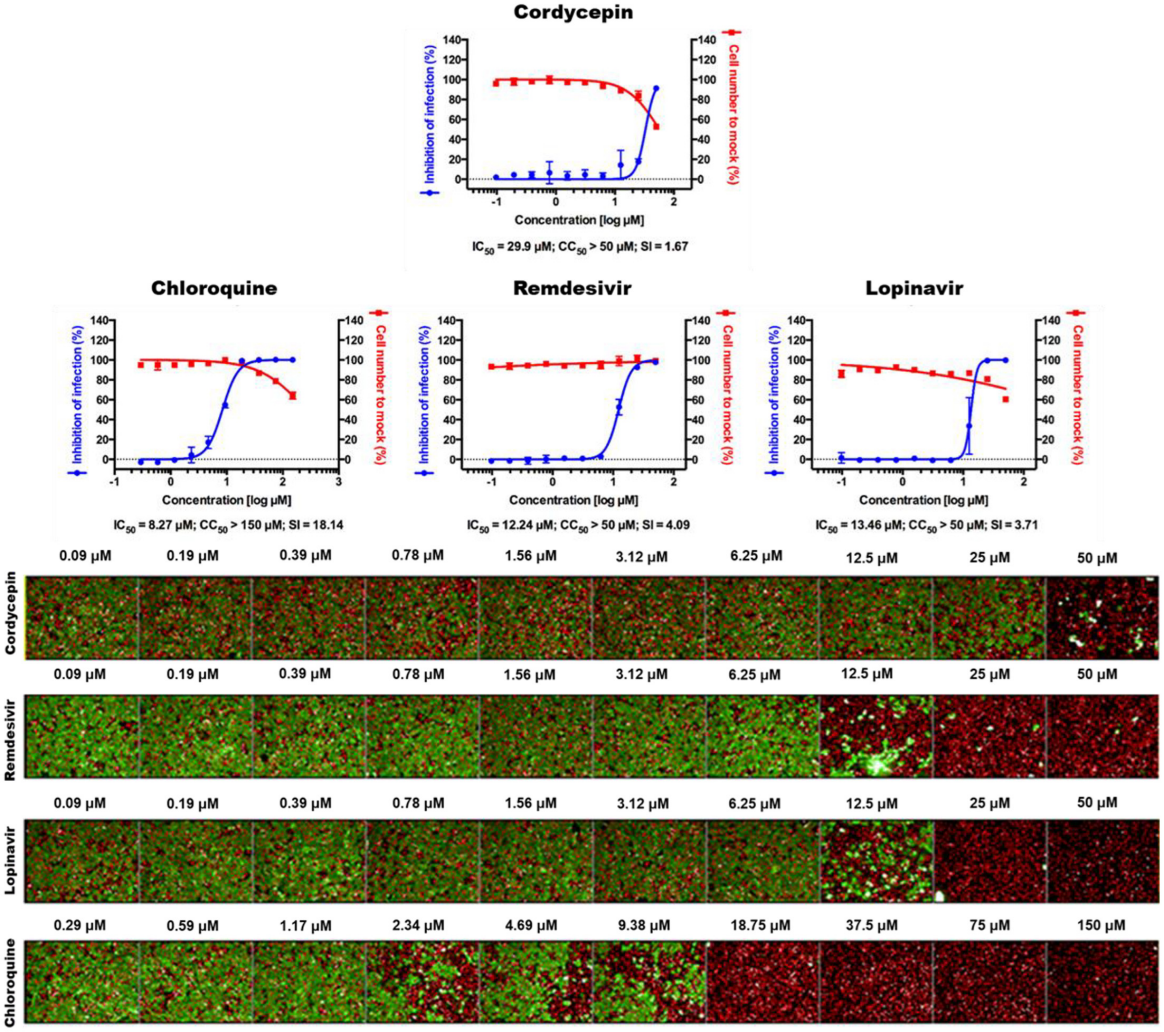
The Dose-response curve (DRC) analysis by immunofluorescence for cordycepin, remdesivir, lopinavir, and chloroquine. The blue circles represent inhibition of SARS-CoV-2 infection (%), and the red squares represent cell viability (%). Means ± SD were calculated from duplicate experiments. The confocal microscope images show cell nuclei (red) and viral N protein (green) at each molecule concentration. The values for half maximum inhibitory concentration (IC_50_), half maximal cytotoxic concentration (CC_50_), and selective index (SI) are shown under each graph.

The potential of cordycepin was further evaluated using SARS-CoV-2 infected Vero cells. Cordycepin and reference drugs were introduced respectively to the cells prior to the viral infection. After 24 h of infection, the cells were evaluated using immunofluorescence analysis using an antibody that targets the viral N protein of SARS-CoV-2. The confocal microscope pictures of both the viral N protein and cell nuclei were examined. Cordycepin showed IC_50_ value of 29 μM whereas a CC_50_ of more than 50 μM. The reference compounds, chloroquine, remdesivir, and lopinavir exhibited IC_50_ values of 8.27, 12.24, and 13.46 μM, respectively (Fig. 9).

The immunofluorescent staining of Vero cells treated with cordycepin clearly shows the inhibition at 50 μM and partial inhibition a 25 μM; as no green staining was observed for Alexa-four-488 tagged viral nucleocapsid protein (Fig. 9). Our findings could be further authenticated using appropriate *in vivo* animal models and other subsequent methods to provide additional therapeutic routes.

## Conclusion

To evaluate *Cordyceps militaris* as a potential candidate for coronaviruses, an analysis of constituents of *C. militaris* against SARS-CoV-2 M^pro^ was conducted by virtual screening analysis. Among ten compounds of *C. militaris*, cordycepin showed the strongest affinity to SARS-CoV-2 M^pro^, an important target for COVID-19 therapeutics. Further analysis of the structure-activity relationship showed the importance of the ribosyl group of nucleoside derivatives and 3’-deoxyribose and 2’-methoxyribose exerted stronger affinity. Cordycepin, which showed the strongest affinity to M^pro^ also bound to M^pro^ mutants. In addition, the binding mechanisms between cordycepin and M^pro^ were further demonstrated by MD simulation, RMSD, and MM/PBSA analysis. PCA analysis also confirmed the conformational change of M^pro^ by cordycepin, which inhibits the function of M^pro^. Finally, the efficacy of cordycepin was evaluated *in vitro* using Vero cells infected with SARS-CoV-2, which showed excellent inhibition with an IC_50_ value of 29 μM.

In summary, Cordyceps mushrooms contain various bioactive components in addition to cordycepin, which were expected to inhibit SARS-CoV-2 replication by binding to M^pro^. Therefore, Cordyceps, with its diverse constituents, is proposed as a potential therapeutic agent for coronavirus. Its use is also economically advantageous, as it can be utilized without the need for further purification. The efficacy of cordycepin has also been confirmed against various SARS-CoV-2 variants, highlighting its broad-spectrum antiviral potential. As a result, *C. militaris* holds promise as an important therapeutic option for coronavirus treatment.

## Figures and Tables

**Table 1:** Comprehensive interaction analysis of compounds within the active site of M^pro^.

Compound	Docking Score	Residue	Distance	Type
Cordycepin (**1**)	-6.774	MET49	2.22	Hydrogen Bond
		HIS164	2.02	Hydrogen Bond
		TYR54	-	Aromatic
		GLU166	-	Charged
		ASP187	-	Charged
Adenosine (**2**)	-6.756	HIS164	1.87	Hydrogen Bond
		TYR54	-	Aromatic
		ASP187	-	Charged
Xanthosine (**3**)	-6.392	GLY143	2.15	Hydrogen Bond
		TYR54	-	Aromatic
		GLU166	-	Charged
2’-*O*-Methyl-adenosine (**4**)	-6.136	ASN142	1.94	Hydrogen Bond
		TYR54	-	Aromatic
		ASP187	-	Charged
Nicotinamide (**5**)	-5.981	HIS164	1.74	Hydrogen Bond
		TYR54	-	Aromatic
		ASP187	-	Charged
Uracil (**6**)	-5.611	HIS164	1.84	Hydrogen Bond
		HIS41	4.00	Pi-Pi Stacking
		ASP187	-	Charged
Uridine (**7**)	-5.493	ASN142	2.17	Hydrogen Bond
		GLU166	2.53	Hydrogen Bond
		HIS41	4.70	Pi-Pi Stacking
		ASP187	-	Charged
2’-Deoxyuridine (**8**)	-5.423	ARG188	2.42	Hydrogen Bond
		TYR54	-	Aromatic
Cordyrrole A (**9**)	-5.394	GLY143	2.07	Hydrogen Bond
		GLN189	2.14	Hydrogen Bond
2-Hydroxy-1-[1-(2-hydroxyethyl)-1*H*-pyrrol-2-yl]-ethan-1-one (**10**)	-5.330	TYR54	1.80	Hydrogen Bond
		GLN189	1.96	Hydrogen Bond
		TYR54	-	Aromatic

**Table 2:** The MM/PBSA binding free energy constituents of cordycepin in complex with M^pro^.

	ΔVDWAALS	ΔEEL	ΔGGAS	ΔGSOLV	ΔTOTAL
Energy Average (kcal/mol)	-28.65	56.99	28.34	-39.03	-10.70
